# Supplementary material for: Enzyme structure correlates with variant effect predictability
Source: Comput Struct Biotechnol J. 2024 Oct 2;23:3489–97. doi: 10.1016/j.csbj.2024.09.007 (PMC11491678; doi:10.1016/j.csbj.2024.09.007)
Supplement: MMC — The supplementary material file provides extra information about the generated and public datasets, like the diversity of the sequences in the generated dataset, the distribution of the number of mutations in the generated dataset and the joint frequencies of mutations occurring at every pair of structural characteristcs in all datasets. Furthermore, plots of Spearman correlation scores on test folds of all datasets are included. [file mmc1.pdf]

A Supplementary Materials

Table S1: Number of variants in the combinatorial dataset per structural class in total and for every number ($1 \leq m \leq 8$) of mutations per variant (e.g. $m = 1$ pertains only to variants with a single mutation).

**Structural characteristic**	**Number of Variants**								
	Total	$m = 1$	$m = 2$	$m = 3$	$m = 4$	$m = 5$	$m = 6$	$m = 7$	$m = 8$
Exposed	1,823	254	519	504	320	168	46	10	2
Buried	1,883	235	528	561	354	142	47	16	0
Few contacts	1,905	232	490	532	374	202	61	12	2
Many contacts	1,801	257	557	533	300	108	32	14	0
Distant to active site	1,924	255	537	574	343	157	45	12	1
Close to active site	1,782	234	510	491	331	153	48	14	1
Part of loop	1,747	248	506	501	313	125	42	11	1
Part of helix or sheet	1,959	241	541	564	361	185	51	15	1

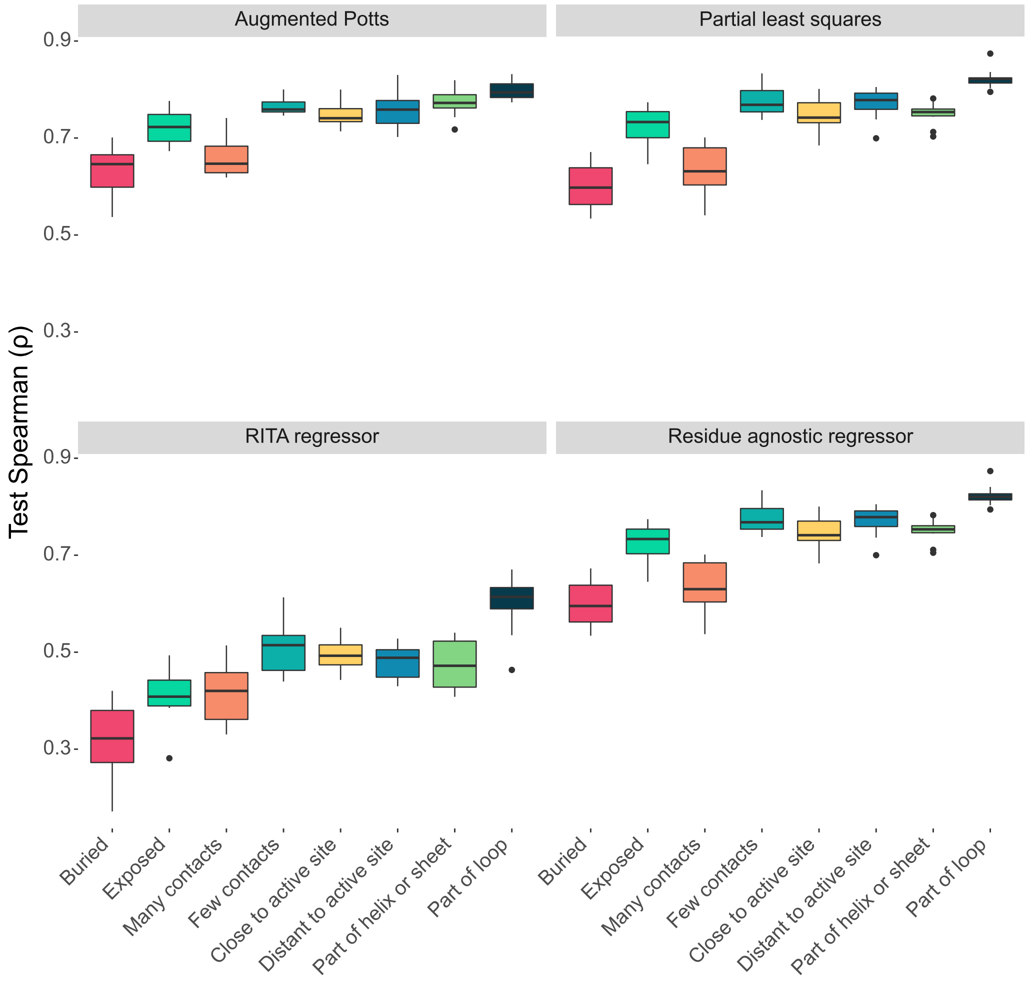

Figure S1: Spearman correlation score distributions of model predictions and ground truths of all models on 10 test folds of the A2RQE2_9CEL dataset. The boxes show the interquartile range (IQR) of test Spearman correlations, with a line indicating the median. Whiskers extend to the smallest and largest values within 1.5 * IQR from the quartiles. Outliers are points outside the whiskers.

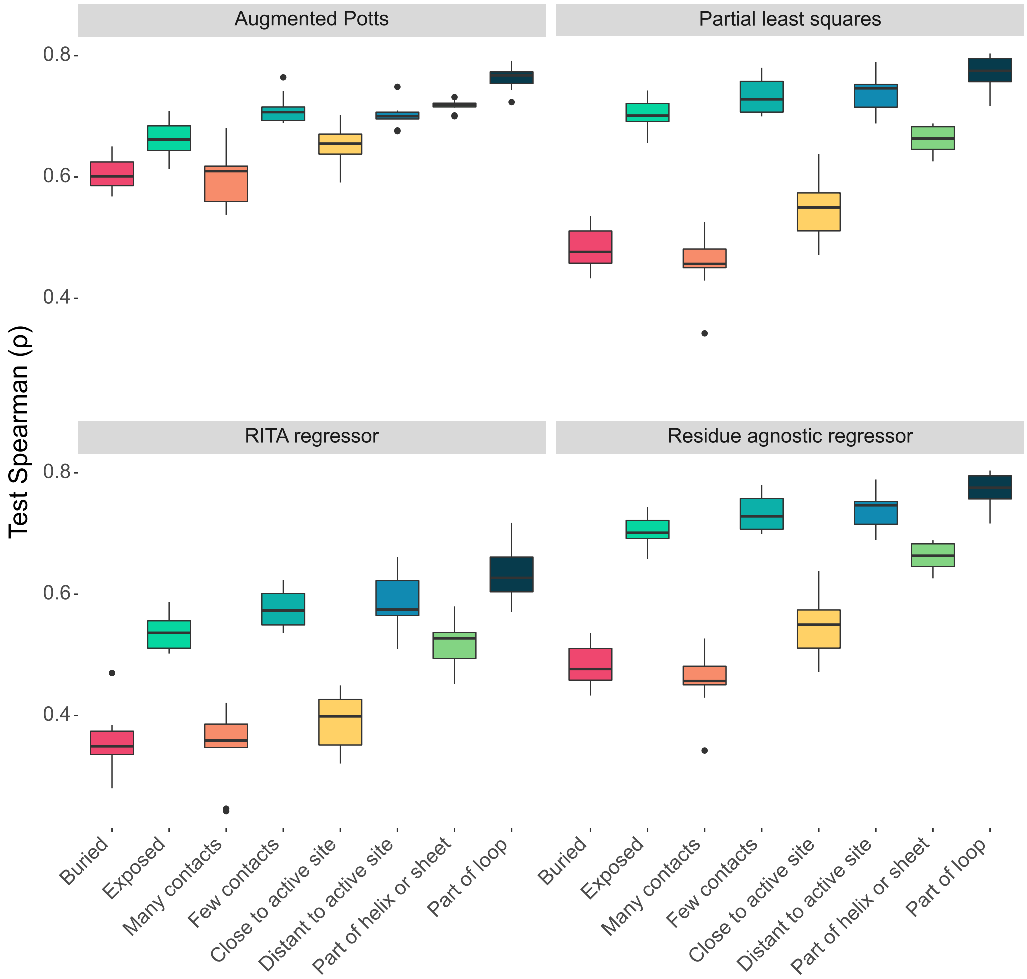

Figure S2: Spearman correlation score distributions of model predictions and ground truths of all models on 10 test folds of the AMIE_PSEAE_Wrenbeck_2017 dataset. The boxes show the interquartile range (IQR) of test Spearman correlations, with a line indicating the median. Whiskers extend to the smallest and largest values within 1.5 * IQR from the quartiles. Outliers are points outside the whiskers.

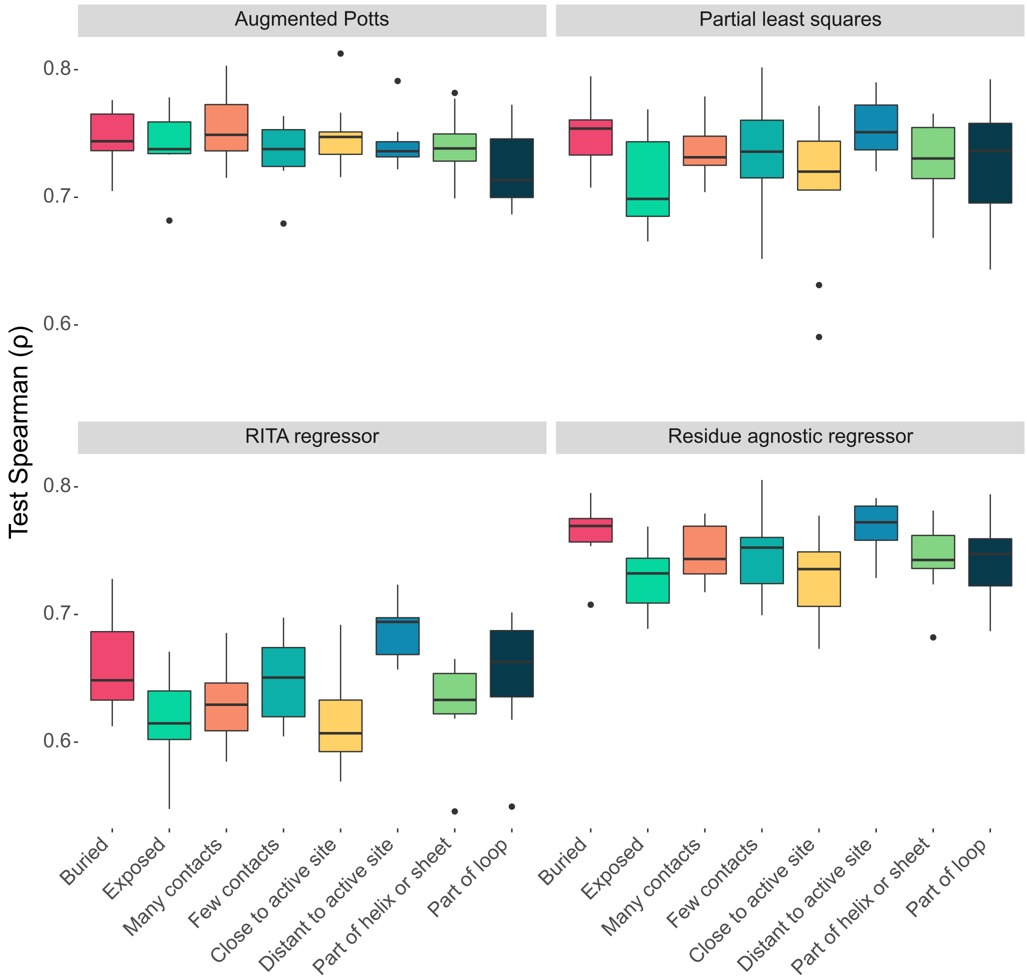

Figure S3: Spearman correlation score distributions of model predictions and ground truths of all models on 10 test folds of the BLAT_ECOLX_Deng_2012 dataset. The boxes show the interquartile range (IQR) of test Spearman correlations, with a line indicating the median. Whiskers extend to the smallest and largest values within 1.5 * IQR from the quartiles. Outliers are points outside the whiskers.

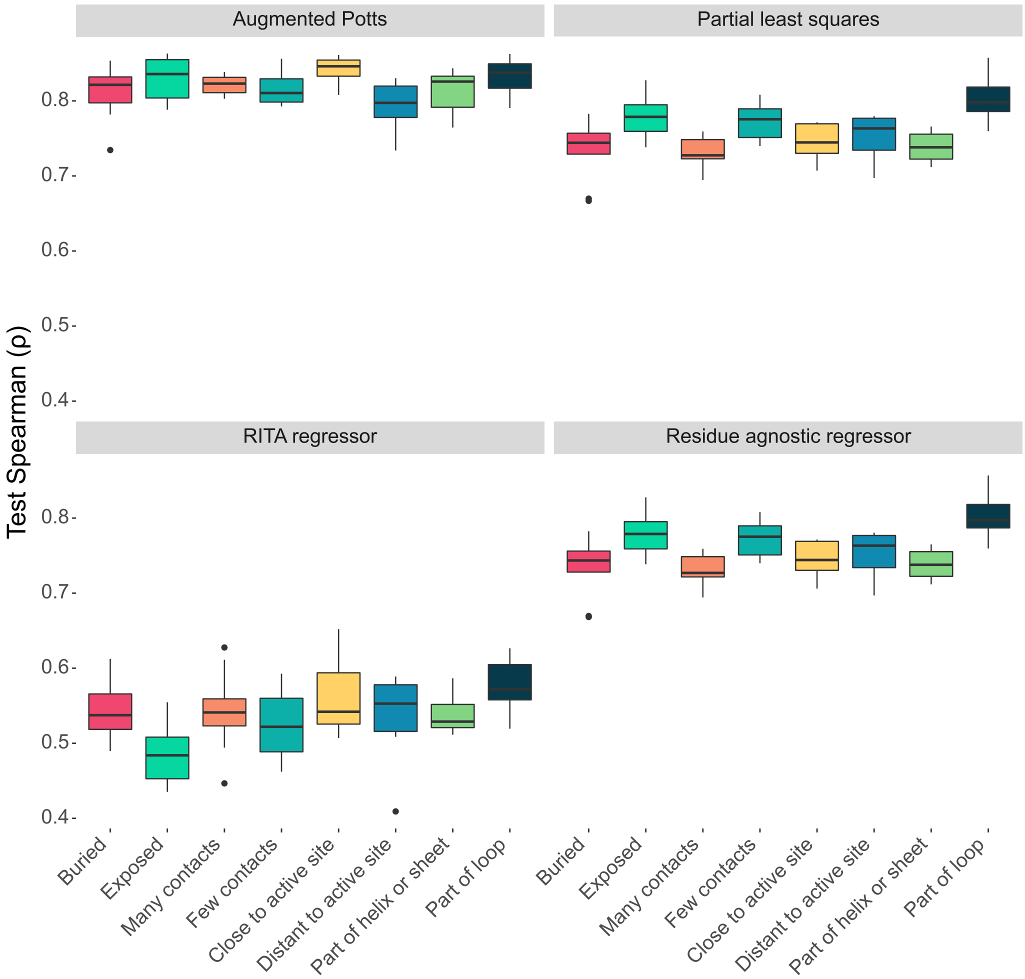

Figure S4: Spearman correlation score distributions of model predictions and ground truths of all models on 10 test folds of the BLAT_ECOLX_Firnberg_2014 dataset. The boxes show the interquartile range (IQR) of test Spearman correlations, with a line indicating the median. Whiskers extend to the smallest and largest values within 1.5 * IQR from the quartiles. Outliers are points outside the whiskers.

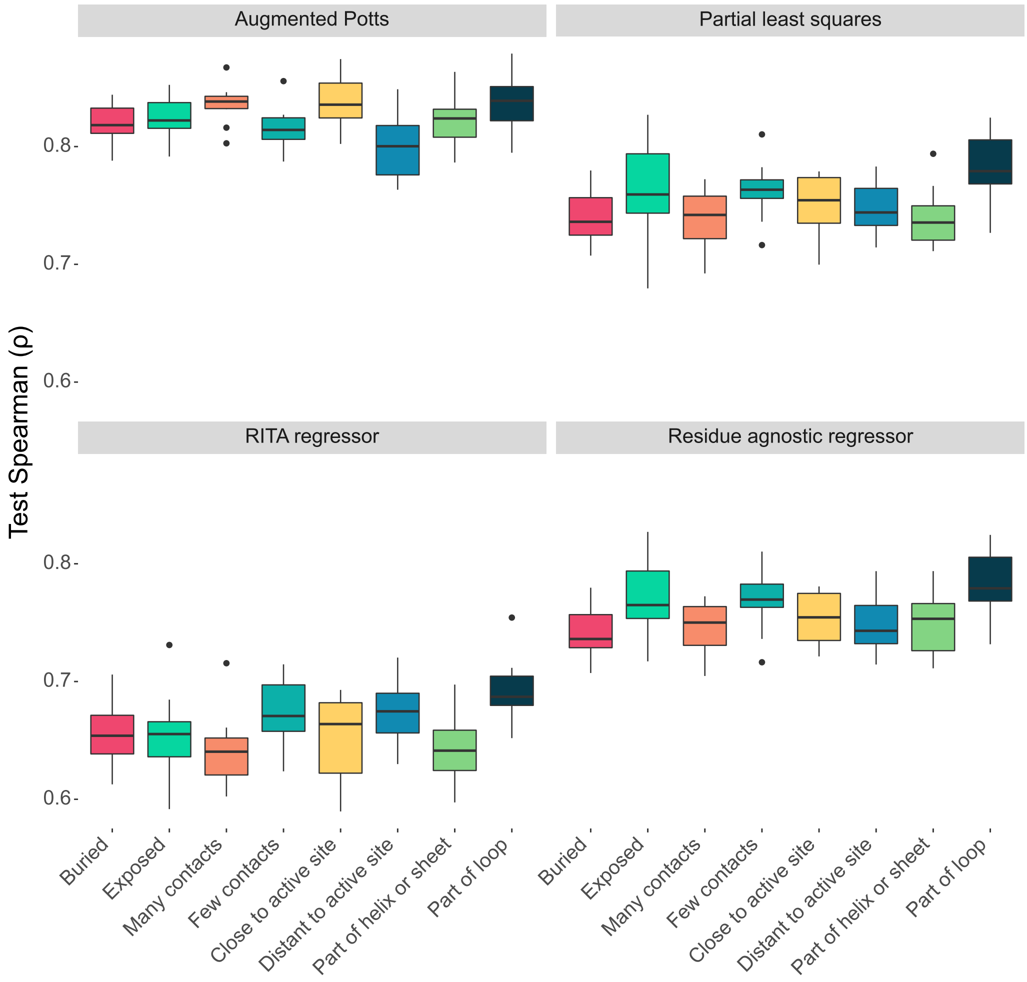

Figure S5: Spearman correlation score distributions of model predictions and ground truths of all models on 10 test folds of the BLAT_ECOLX_Stiffler_2015 dataset. The boxes show the interquartile range (IQR) of test Spearman correlations, with a line indicating the median. Whiskers extend to the smallest and largest values within 1.5 * IQR from the quartiles. Outliers are points outside the whiskers.

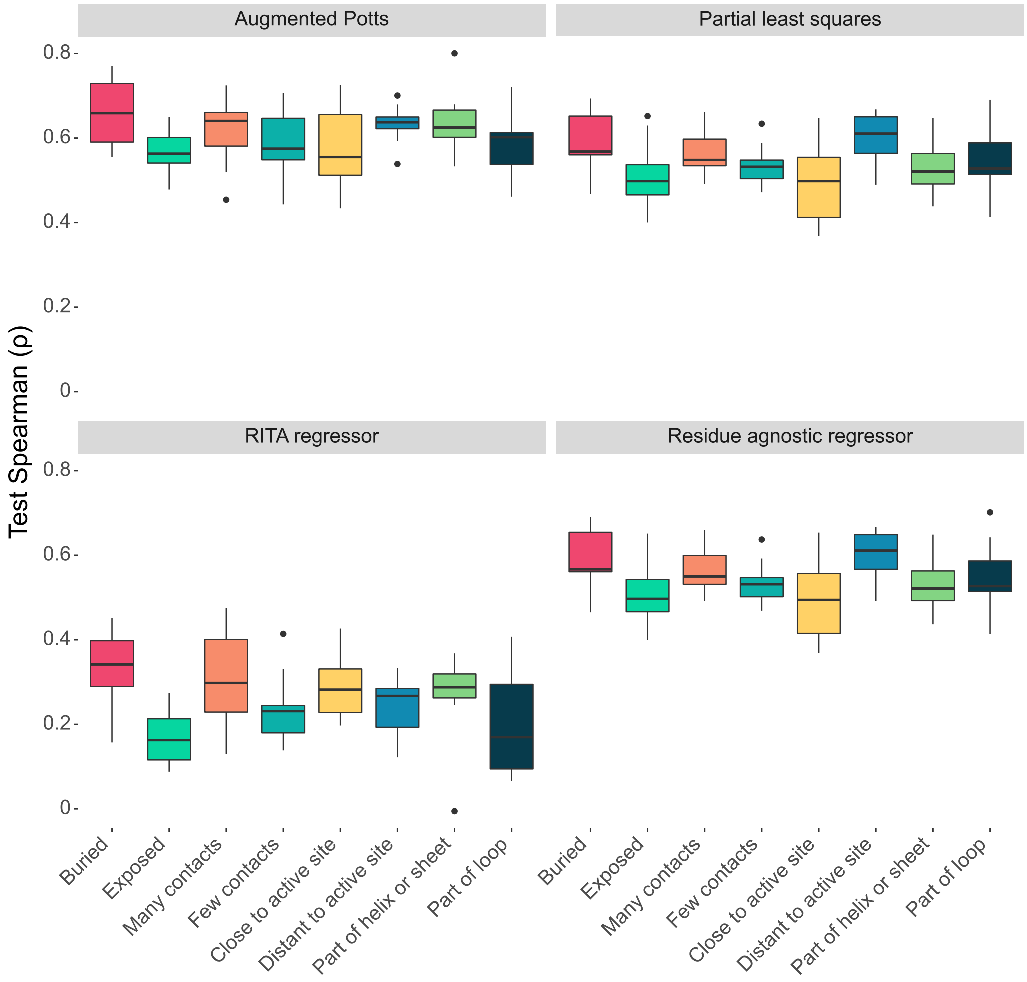

Figure S6: Spearman correlation score distributions of model predictions and ground truths of all models on 10 test folds of the CASP3_HUMAN_Roychowdhury_2020 dataset. The boxes show the interquartile range (IQR) of test Spearman correlations, with a line indicating the median. Whiskers extend to the smallest and largest values within 1.5 * IQR from the quartiles. Outliers are points outside the whiskers.

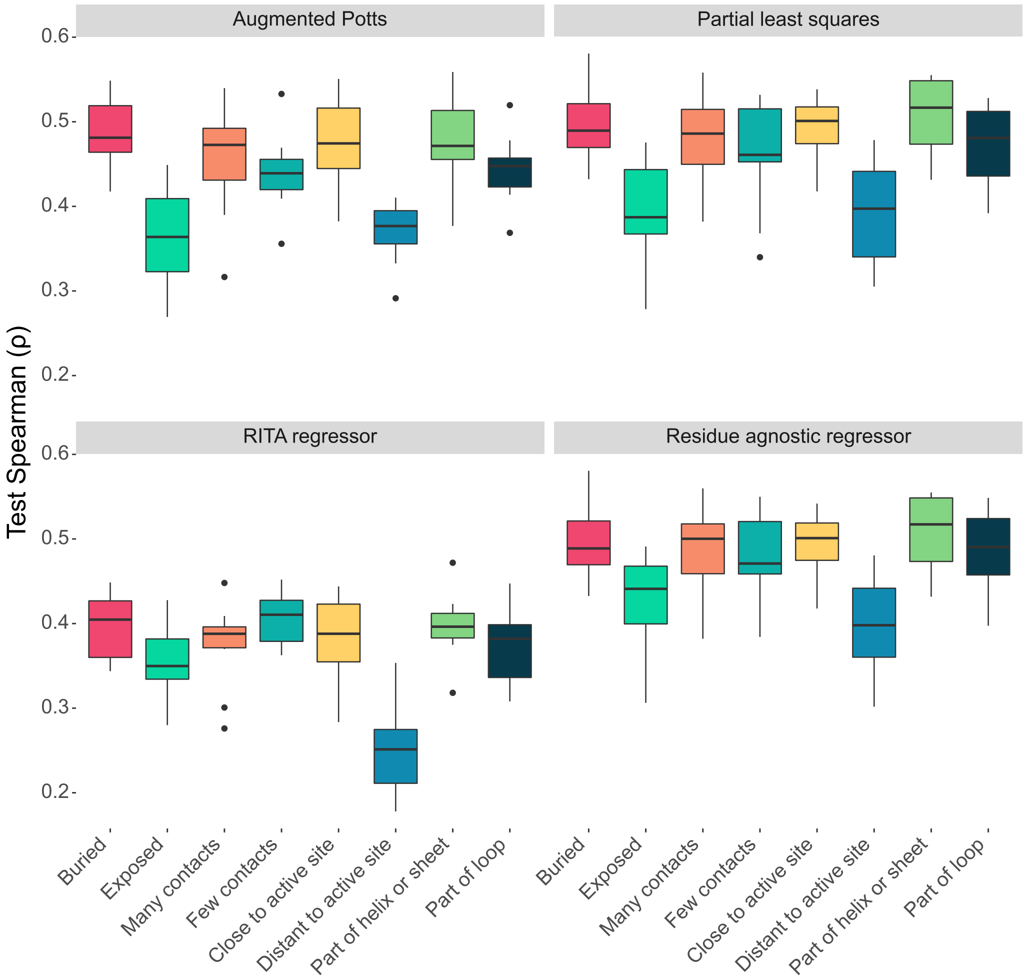

Figure S7: Spearman correlation score distributions of model predictions and ground truths of all models on 10 test folds of the MK01_HUMAN_Brenan_2016 dataset. The boxes show the interquartile range (IQR) of test Spearman correlations, with a line indicating the median. Whiskers extend to the smallest and largest values within 1.5 * IQR from the quartiles. Outliers are points outside the whiskers.

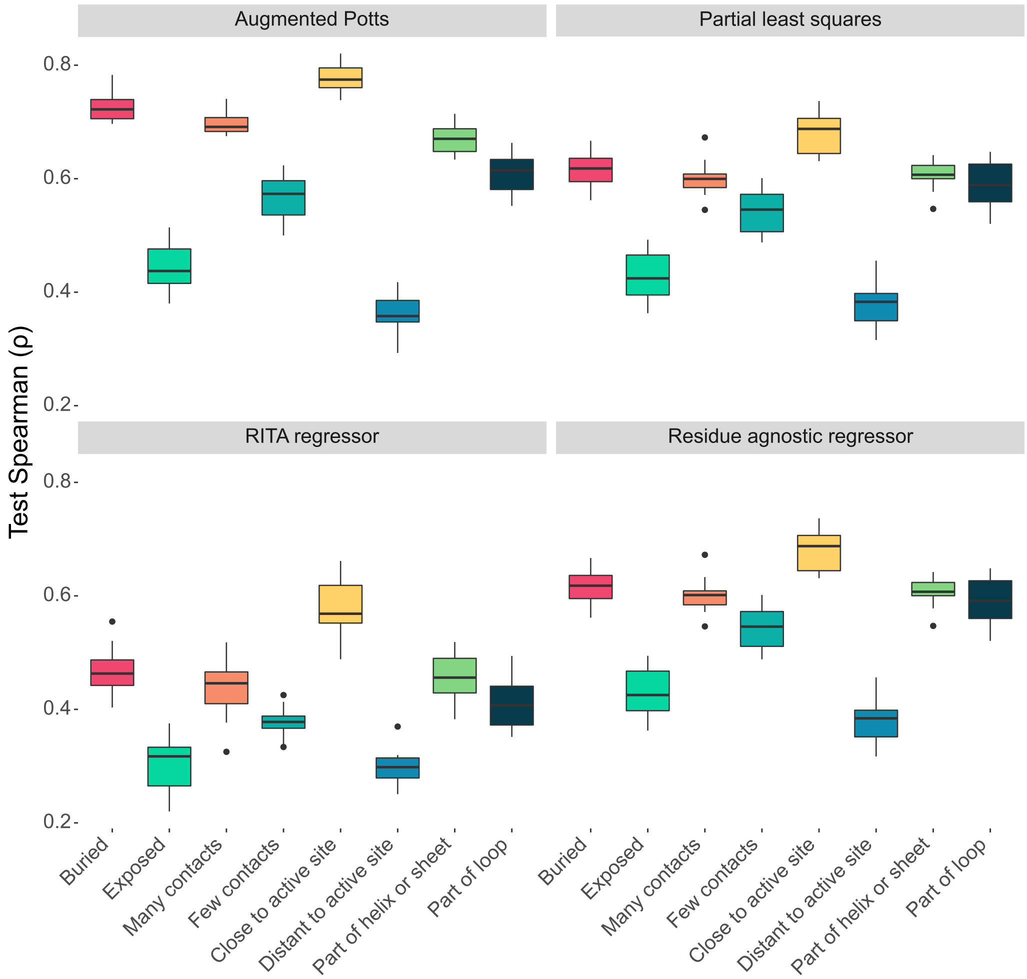

Figure S8: Spearman correlation score distributions of model predictions and ground truths of all models on 10 test folds of the PTEN_HUMAN_Mighell_2018 dataset. The boxes show the interquartile range (IQR) of test Spearman correlations, with a line indicating the median. Whiskers extend to the smallest and largest values within 1.5 * IQR from the quartiles. Outliers are points outside the whiskers.

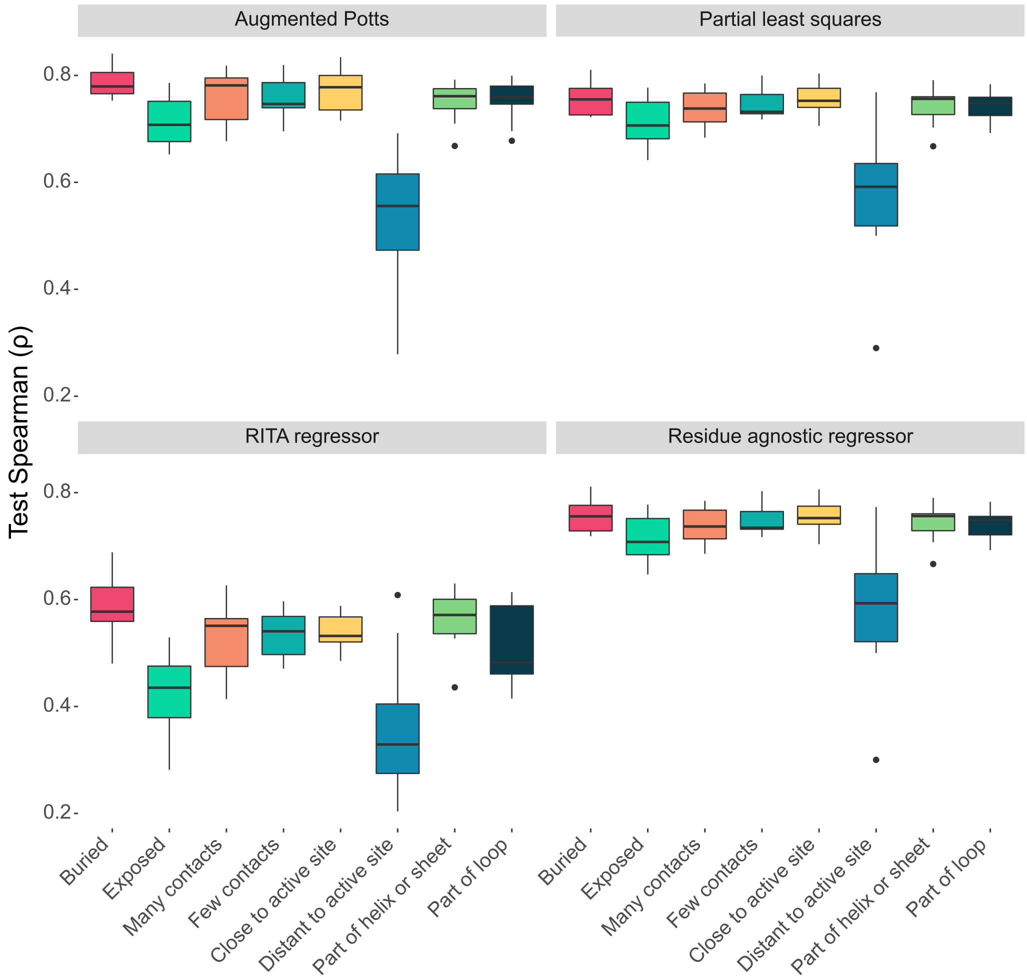

Figure S9: Spearman correlation score distributions of model predictions and ground truths of all models on 10 test folds of the SRC_HUMAN_Ahler_2019 dataset. The boxes show the interquartile range (IQR) of test Spearman correlations, with a line indicating the median. Whiskers extend to the smallest and largest values within 1.5 * IQR from the quartiles. Outliers are points outside the whiskers.

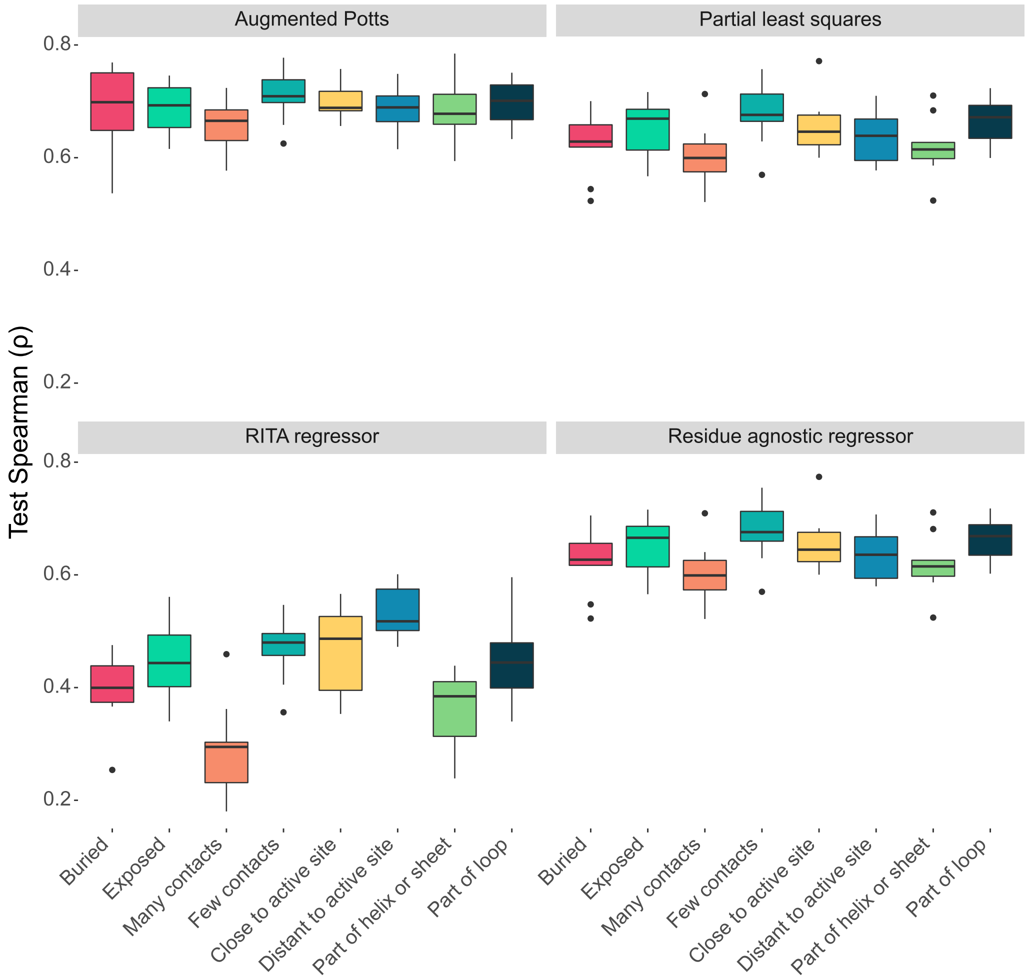

Figure S10: Spearman correlation score distributions of model predictions and ground truths of all models on 10 test folds of the UBC9_HUMAN_Weile_2017 dataset. The boxes show the interquartile range (IQR) of test Spearman correlations, with a line indicating the median. Whiskers extend to the smallest and largest values within 1.5 * IQR from the quartiles. Outliers are points outside the whiskers.

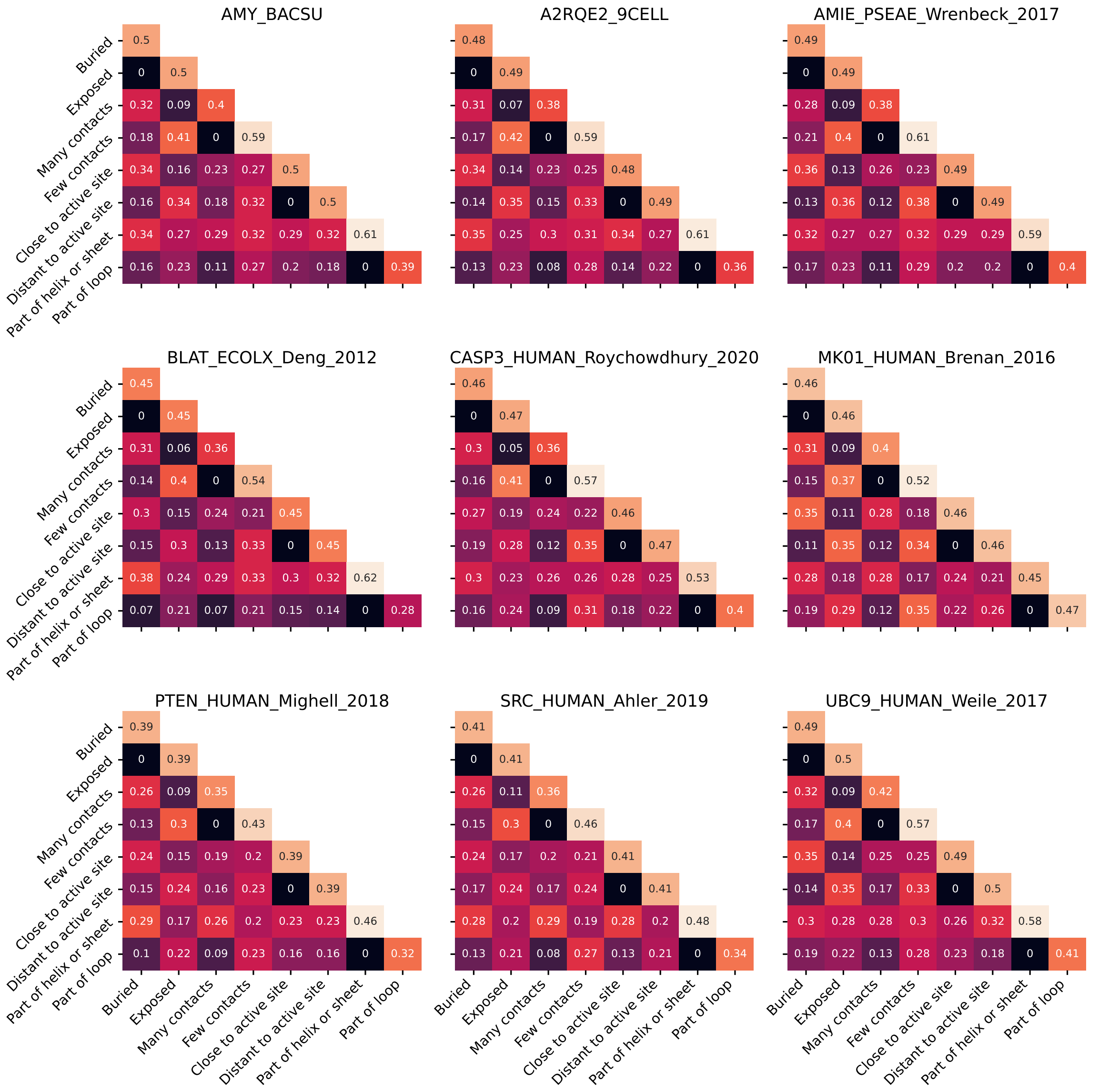

Figure S11: Joint frequencies of residue membership for every pair of structural classes of all enzymes, *i.e.*, the frequencies of structurally resolved residues simultaneously being a member of structural class C_i and structural class C_j.

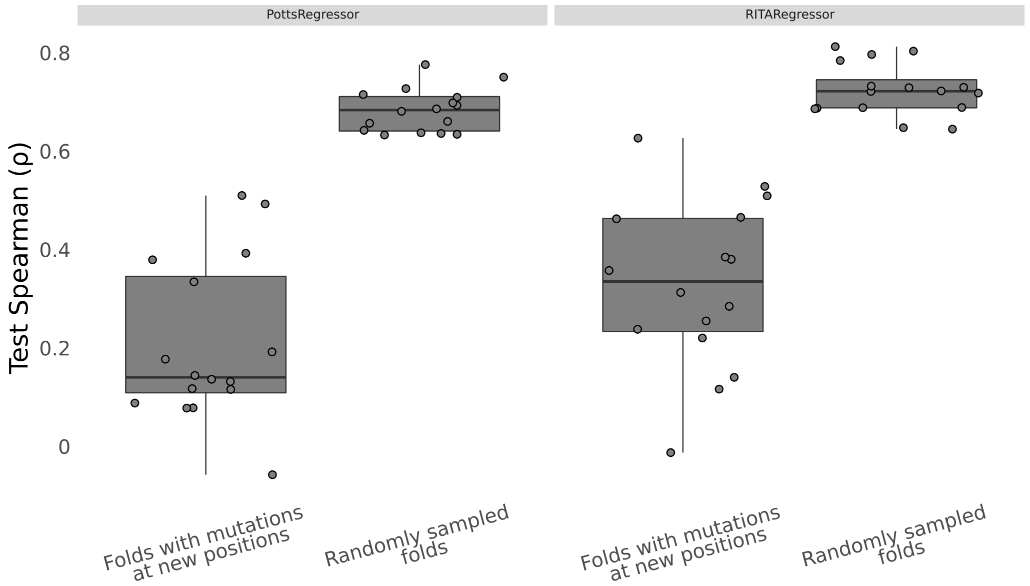

Figure S12: Test Spearman correlations of the augmented Potts model and RITA regressor over 16 different folds of the combinatorial variant data. Left (“Folds with mutations at novel positions”): performance on variants with mutations at positions not observed in the training data. Right (“Randomly sampled folds”): performances of models on randomly split folds.

Table S2: Titles of the studies corresponding to the public datasets used in our analysis.

Dataset name	Study
AMIE_PSEAE_Wrenbeck_2017	Single-mutation fitness landscapes for an enzyme on multiple substrates reveal specificity is globally encoded (1)
BLAT_ECOLX_Deng_2012	Deep Sequencing of Systematic Combinatorial Libraries Reveals β-Lactamase Sequence Constraints at High Resolution (2)
BLAT_ECOLX_Firnberg_2014	A Comprehensive, High-Resolution Map of a Gene's Fitness Landscape (3)
BLAT_ECOLX_Stiffler_2015	Evolvability as a Function of Purifying Selection in TEM-1 β-lactamase (4)
CASP3_HUMAN_Roychowdhury_2020	Microfluidic deep mutational scanning of the human executioner caspases reveals differences in structure and regulation (5)
MK01_HUMAN_Brenan_2016	Phenotypic Characterization of a Comprehensive Set of MAPK1 /ERK2 Missense Mutants (6)
PTEN_HUMAN_Mighell_2018	A Saturation Mutagenesis Approach to Understanding PTEN Lipid Phosphatase Activity and Genotype-Phenotype Relationships (7)
SRC_HUMAN_Ahler_2019	A Combined Approach Reveals a Regulatory Mechanism Coupling Src's Kinase Activity, Localization, and Phosphotransferase-Independent Functions (8)
UBC9_HUMAN_Weile_2017	A framework for exhaustively mapping functional missense variants (9)

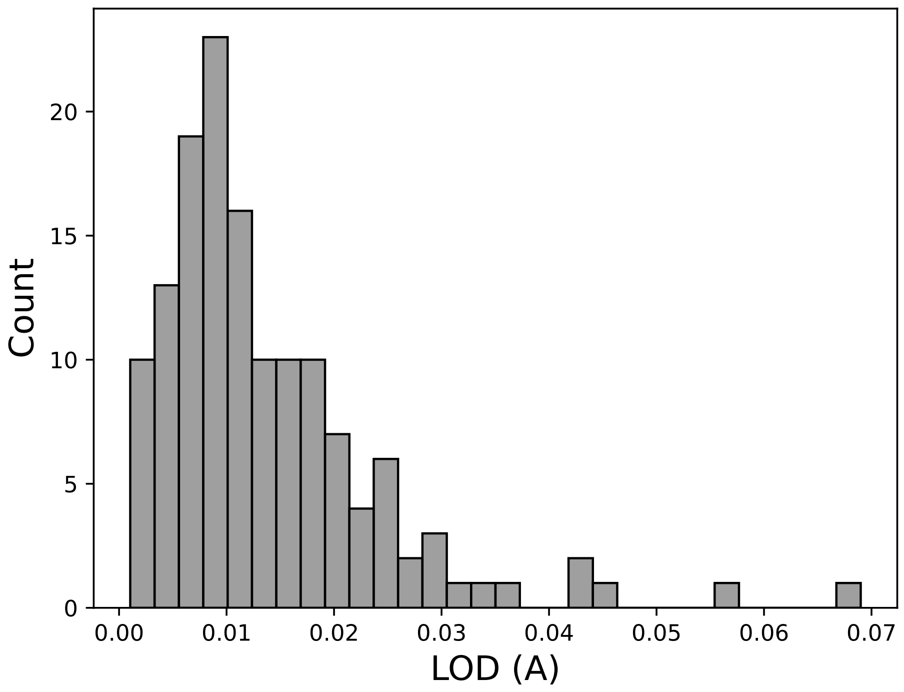

Figure S13: A histogram of the estimates of the limit of detection (LODs) of each of the 141 plates. Each LOD estimate was calculated using the two blank absorbances as well as the absorbance of a zero concentration enzyme solution belonging to the standard calibration curve, which is identical to the blank solution.

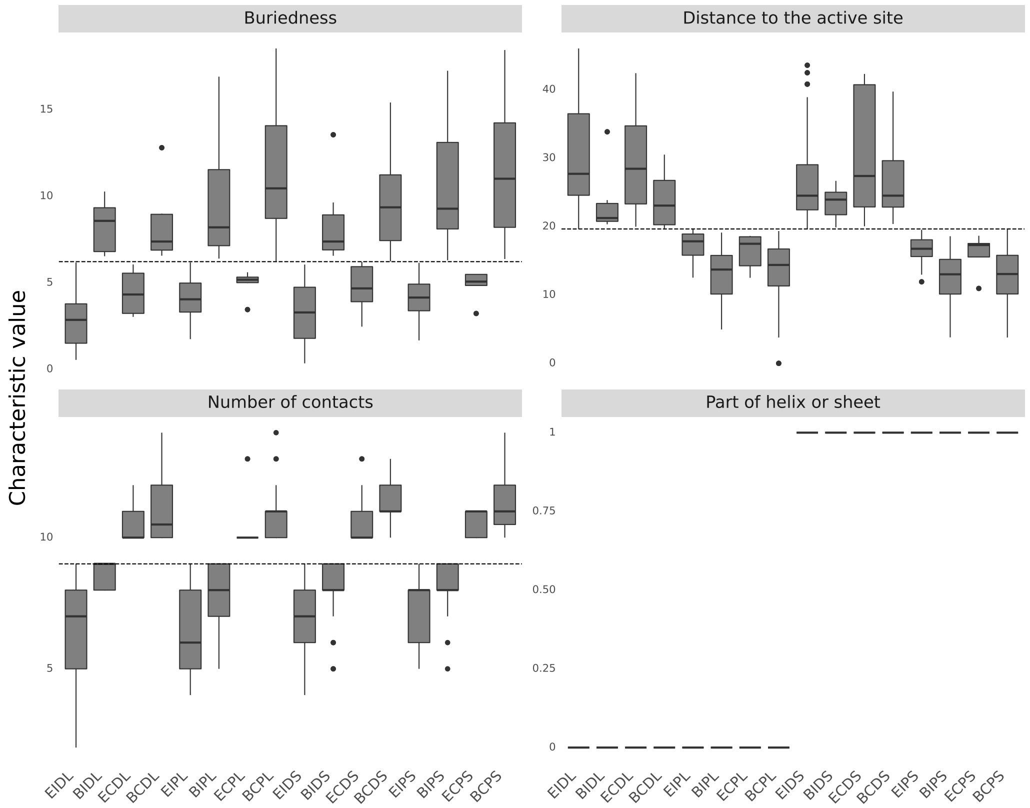

Figure S14: The calculated values of all structural characteristics of residues belonging to each bin. Individual bins are labeled with a four letter code specifying structural class membership. Reading from left to right, letters stand for B: Buried or E: Exposed, C: Closely connected or I: Loosely connected, P: Close to the active site or D: Distant to the active site and S: Part of helix or sheet or L: Part of loop. The median value used as threshold in assigning binary labels is visualized with the dashed line. For structural characteristics "Buriedness", "Distance to the active site" and "Number of contacts", bins with values below the median contain positions where residues are buried, are close to the active site and have few contact residues, respectively.

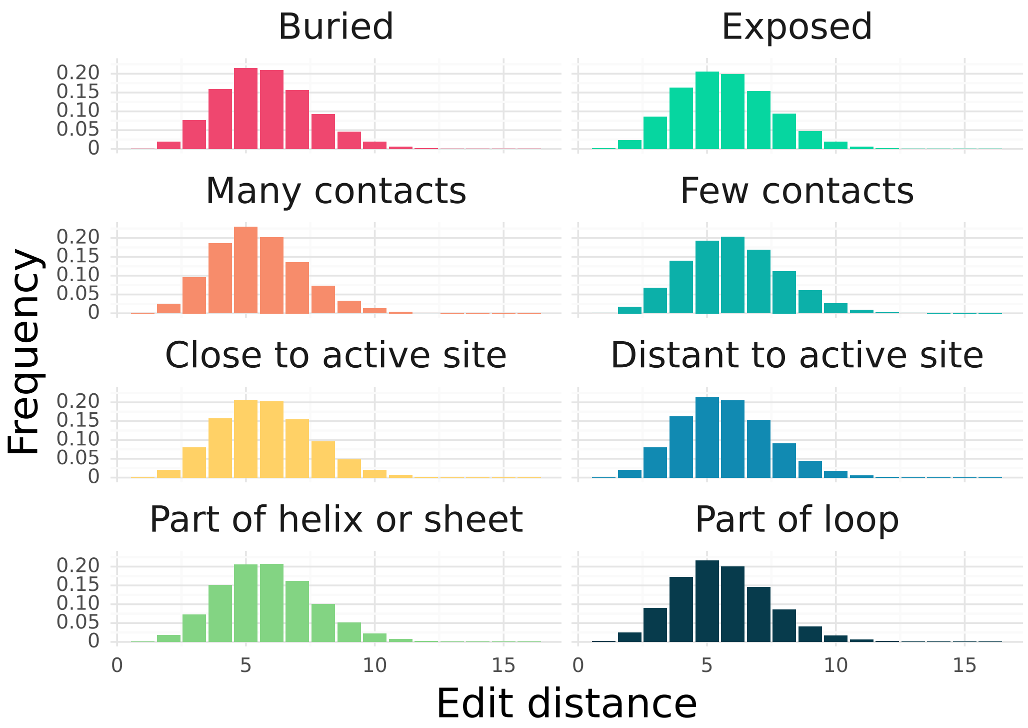

Figure S15: Diversity of the variants in each structural class presented as the distributions of all versus all edit distances within each structural class. Nearly identical distributions of edit distances demonstrate that the degree of variant diversity does not vary significantly among structural classes.

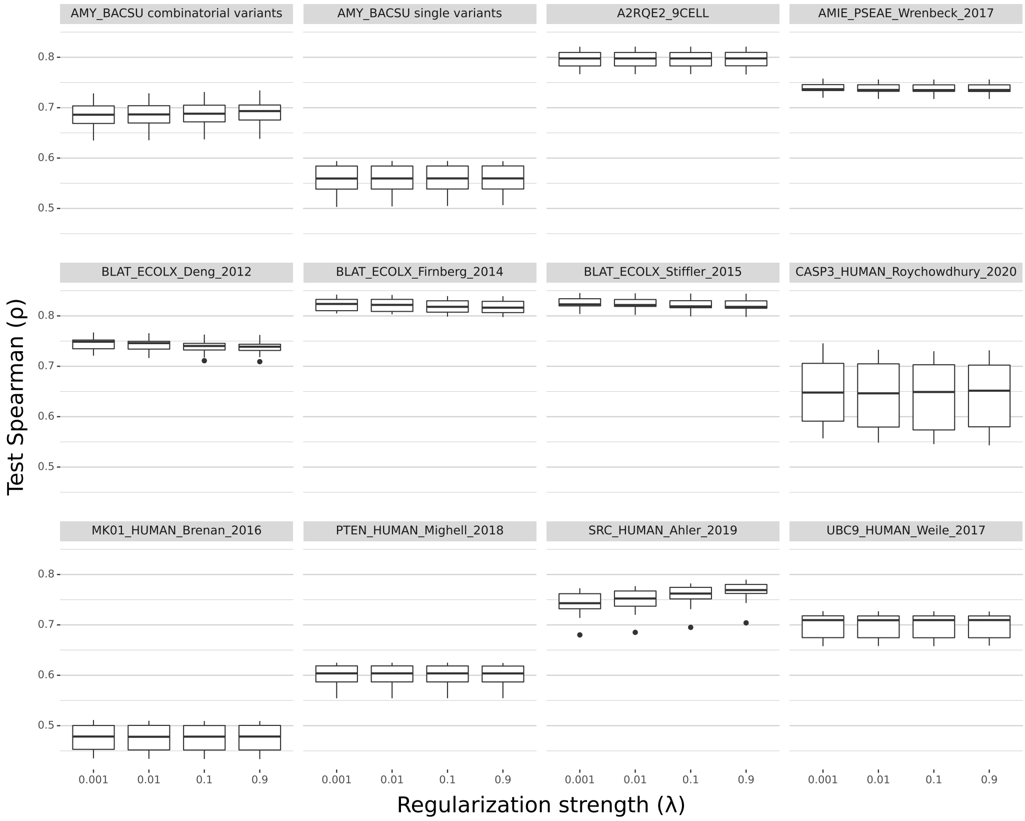

Figure S16: The effect of regularization strength on augmented Potts performance. Test Spearman scores are obtained from 10-fold cross validation of the model on each dataset in its entirety.

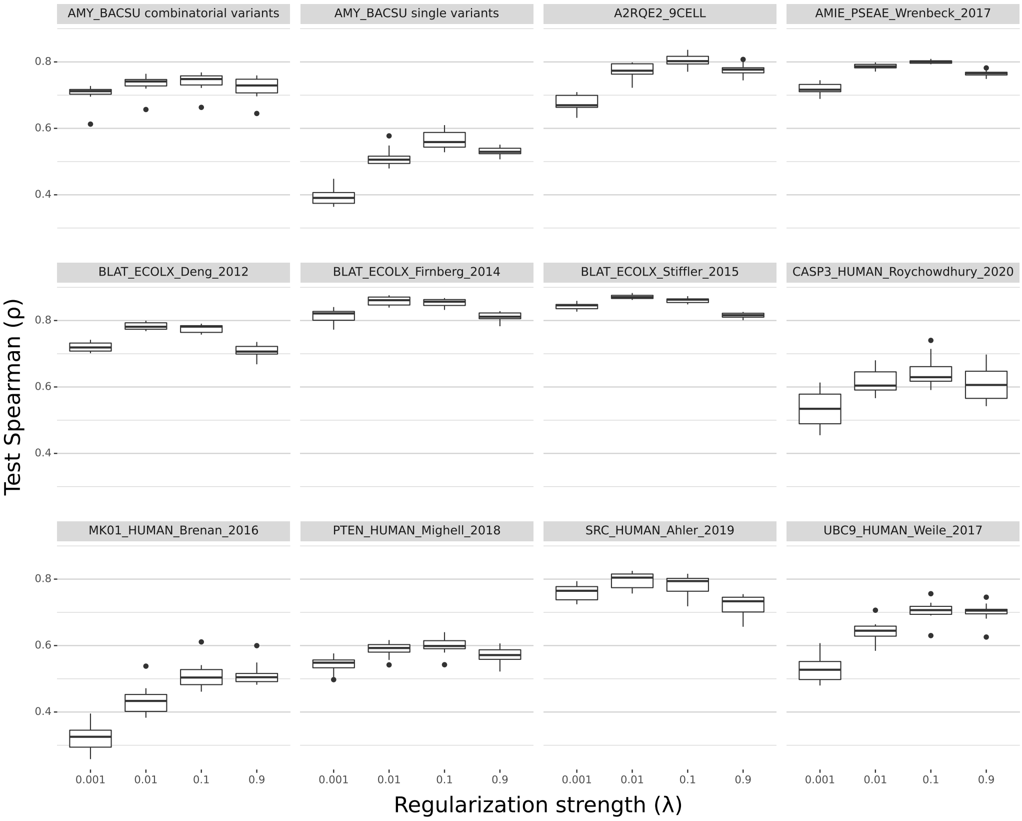

Figure S17: The effect of regularization strength on RITA regressor performance. Test Spearman scores are obtained from 10-fold cross validation of the model on each dataset in its entirety.

References

[1] Wrenbeck, E. E., Azouz, L. R. & Whitehead, T. A. Single-mutation fitness landscapes for an enzyme on multiple substrates reveal specificity is globally encoded. *Nature communications* **8**, 15695 (2017).

[2] Deng, Z. *et al.* Deep sequencing of systematic combinatorial libraries reveals β-lactamase sequence constraints at high resolution. *Journal of molecular biology* **424**, 150–167 (2012).

[3] Firnberg, E., Labonte, J. W., Gray, J. J. & Ostermeier, M. A comprehensive, high-resolution map of a gene's fitness landscape. *Molecular biology and evolution* **31**, 1581–1592 (2014).

[4] Stiffler, M. A., Hekstra, D. R. & Ranganathan, R. Evolvability as a function of purifying selection in TEM-1 β-lactamase. *Cell* **160**, 882–892 (2015).

[5] Roychowdhury, H. & Romero, P. A. Microfluidic deep mutational scanning of the human executioner caspases reveals differences in structure and regulation. *Cell Death Discovery* **8**, 7 (2022).

[6] Brenan, L. *et al.* Phenotypic characterization of a comprehensive set of MAPK1/ERK2 missense mutants. *Cell reports* **17**, 1171–1183 (2016).

[7] Mighell, T. L., Evans-Dutson, S. & O'Roak, B. J. A saturation mutagenesis approach to understanding PTEN lipid phosphatase activity and genotype-phenotype relationships. *The American Journal of Human Genetics* **102**, 943–955 (2018).

[8] Ahler, E. *et al.* A combined approach reveals a regulatory mechanism coupling Src's kinase activity, localization, and phosphotransferase-independent functions. *Molecular cell* **74**, 393–408 (2019).

[9] Weile, J. *et al.* A framework for exhaustively mapping functional missense variants. *Molecular systems biology* **13**, 957 (2017).
